# Detection of SARS-CoV-2 lineage P.1 in patients from a region with exponentially increasing hospitalisation rate, February 2021, Rio Grande do Sul, Southern Brazil

**DOI:** 10.2807/1560-7917.ES.2021.26.12.2100276

**Published:** 2021-03-25

**Authors:** Andreza Francisco Martins, Alexandre Prehn Zavascki, Priscila Lamb Wink, Fabiana Caroline Zempulski Volpato, Francielle Liz Monteiro, Clévia Rosset, Fernanda De-Paris, Álvaro Krüger Ramos, Afonso Luís Barth

**Affiliations:** 1 LABRESIS – Laboratório de Pesquisa em Resistência Bacteriana, Hospital de Clínicas de Porto Alegre, Porto Alegre, Rio Grande do Sul, Brazil; 2 Programa de Pós-Graduação em Ciências Farmacêuticas, Universidade Federal do Rio Grande do Sul, Porto Alegre, Rio Grande do Sul, Brazil; 3 Bioinformatic Core, Hospital de Clínicas de Porto Alegre, Porto Alegre, Rio Grande do Sul, Brazil; 4 These authors contributed equally to the work as first authors.; 5 Internal Medicine Department, Universidade Federal do Rio Grande do Sul, Porto Alegre, Brazil; 6 Infectious Diseases Service, Hospital de Clínicas de Porto Alegre, Porto Alegre, Rio Grande do Sul, Brazil; 7 Laboratório de Diagnóstico de SARS-CoV-2, Hospital de Clínicas de Porto Alegre, Porto Alegre, Rio Grande do Sul, Brazil; 8 Programa de Pós-Graduação em Ciências Médicas, Universidade Federal do Rio Grande do Sul, Porto Alegre, Rio Grande do Sul, Brazil; 9 Laboratório de Medicina Genômica (LMG), Hospital de Clínicas de Porto Alegre, Porto Alegre, Rio Grande do Sul, Brazil; 10 Programa de Pós-Graduação em Matemática, Universidade Federal do Rio Grande do Sul, Porto Alegre, Rio Grande do Sul, Brazil

**Keywords:** SARS-CoV-2, P.1 lineage, COVID-19

## Abstract

The emergence of SARS-CoV-2 P.1 lineage coincided with a surge in hospitalisations in the North region of Brazil. In the South region’s Rio Grande do Sul state, severe COVID-19 case numbers rose 3.8 fold in February 2021. During that month, at a COVID-19 referral hospital in this state, whole-genome sequencing of a subset of cases’ specimens (n = 27) revealed P.1 lineage SARS-CoV-2 in most (n = 24). Findings raise concerns regarding a possible association between lineage P.1 and rapid case and hospitalisation increases.

From epidemiological week 6, starting on 7 February 2021, until 6 March, the number of hospitalisations for coronavirus disease (COVID-19) in Rio Grande do Sul (RS), the southernmost state of Brazil in the South region, increased from 1,738 inpatients to 6,995 (3.8-fold) [1]. This resulted in the collapse of the state healthcare system [1]. Community transmission of severe acute respiratory syndrome coronavirus 2 (SARS-CoV-2) P.1 lineage was documented in a highly visited RS municipality 1 week before week 6 [2]. We thus investigated whether the P.1 variant of concern (VOC) could be detected in patient specimens from February 2021 in a large tertiary care hospital (830 beds) in RS, which serves also as a COVID-19 referral centre in the state capital, Porto Alegre. We also tested specimens obtained in January 2021 to compare with our February 2021 findings, as well as some from the previous year.

## COVID-19 cases, hospitalisations and fatalities

**Figure 1:**
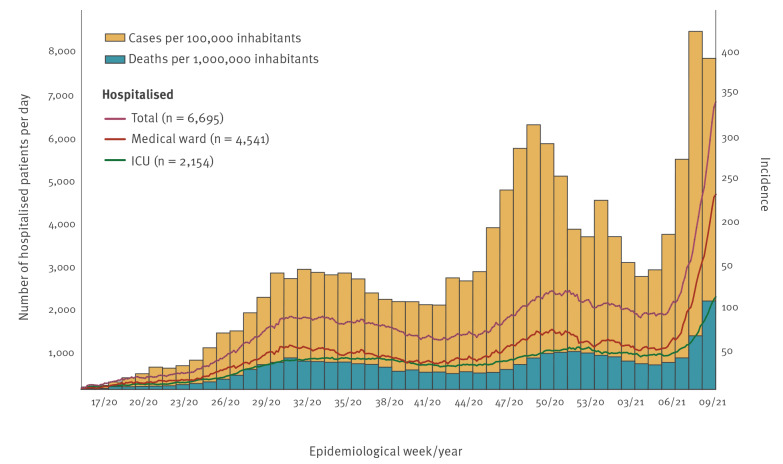
Number of hospitalised COVID-19 cases, as well as incidence of COVID-19 cases and deaths associated to COVID-19 in Rio Grande do Sul state, Southern Brazil, 12 April 2020–6 March 2021 (n = 6,695 hospitalised cases)

COVID-19: coronavirus disease; ICU: intensive care unit.

A COVID-19 case was defined as a person with a positive diagnostic test (reverse transcription quantitative – RT-qPCR) for SARS-CoV-2. From epidemiological week 6 2021 through week 9 2021, the total number of hospitalised COVID-19 cases in RS state exhibited exponential growth, with an average doubling time of 13.4 days and a daily growth rate of 5.3% (Figure 1) [1]. In the same period, a similar exponential growth (doubling time 13.3 days, daily growth rate 5.4%) was observed for hospitalisations in the city of Porto Alegre, where most P.1 specimens were found in the current study (not shown in Figure 1). It is noteworthy that case and death counts for epidemiological weeks 8 2021 and 9 2021 are not final and are subject to increase due to the lag in reporting.

## Sampling and sequencing of COVID-19 cases’ specimens 

A total of 337 and 307 oro/nasopharyngeal swab specimens were obtained from hospitalised cases and outpatient cases in February 2021 and January 2021 respectively. Of these, 27 (8.0%) from February and 15 (4.9%) from January were selected for genomic analysis based on lower cycle threshold (Ct) values (less than 25) of the RT-qPCR assay [3] and patient age (specimens from cases younger than 40 years old were preferred). For 2021, specimens from cases younger than 40 years of age were preferred, because our impression was that there could be a shift to younger age groups being affected and we expected to detect the P.1 variant. Of 3,224 specimens obtained from in- and outpatient cases in 2020, 26 (0.8%) were randomly selected among those with lower Ct values.

Sequencing libraries were prepared using the CleanPlex SARS-CoV-2 panel (Paragon Genomics, Hayward, United States (US)) protocol for target enrichment and library preparation, following manufacturer instructions (https://www.paragongenomics.com/wp-content/uploads/2020/03/UG4001-01_-CleanPlex-SARS-CoV-2-Panel-User-Guide.pdf). The resulting libraries were pooled in equimolar amounts and sequenced in Illumina MiSeq (Illumina, San Diego, US). Consensus sequences were generated by the QIASeq SARS-CoV-2 pipeline (QIAGEN CLC Genomics Workbench 21, Germantown, US). We obtained 68 whole genome sequences of high quality (coverage > 80%, < 10% N, > 29.5 Kb). The lineages were classified using Phylogenetic Assignment of Named Global Outbreak LINeages (Pangolin) [4]. The sequences were deposited into the GISAID database (https://www.gisaid.org/).

## Ethical statement

This study was approved by the Ethics Committees from Hospital de Clínicas de Porto Alegre (CAAE: 30767420.2.0000.5327).

## SARS-CoV-2 lineages identified in cases’ specimens

The specimens obtained in February 2021 (n = 27; Ct value range: 11.08–24.62) belonged to cases aged from 8 months to 62 years; 11 were male. Specimens from January 2021 (n = 15; Ct value range: 9.80–19.32) originated from cases aged 2 months to 58 years, four of whom were male, while those from 2020 (n = 26; Ct value range: 9.39–24.93) were derived from cases between 30 and 82 years of age, 10 male (Supplementary Table).

Among the 68 total specimens, which were all whole genome sequenced, 43 (63.2%) and 25 (36.8%) were from outpatients and inpatients, respectively; there was no significant difference in proportion of the specimen recovery setting across the periods of study (p = 0.46; chi-squared test).

Of the 27 specimens from February 2021, 24 were identified as lineage P.1 compared with one of 15 specimens obtained in January 2021 and none among the 26 specimens in 2020. Age groups of the cases with P.1 lineage were: 20–29 years old (n = 13); 30–39 (n = 4); 40–49 (n = 2); 50–59 (n = 3); 60–69 (n = 1) for adults; and under 2 years (n = 2) for children.

**Figure 2:**
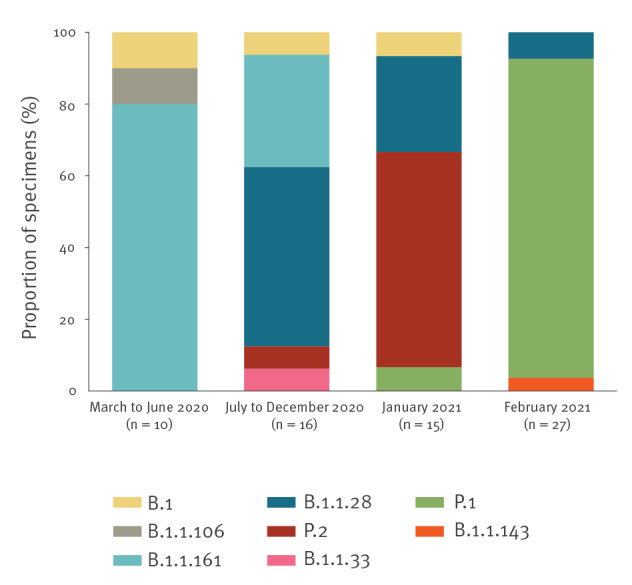
Distribution of SARS-CoV-2 lineages across specimens from COVID-19 cases collected during distinct periods of the study, Rio Grande do Sul state, Southern Brazil, March 2020–February 2021 (n = 68 specimens)

COVID-19: coronavirus disease; SARS-CoV-2: severe acute respiratory syndrome coronavirus 2.

In the other three specimens from February 2021, SARS-CoV-2 lineages B.1.1.28 (n = 2) and B.1.1.143 (n = 1) were found. The lineages identified in January and February 2021 and throughout 2020 are represented in Figure 2.

March–June 2020: B.1 (n = 1 specimen), B.1.1.106 (n = 1), and B.1.1.161 (n = 8); July–December 2020: B.1 (n = 1), B.1.1.161 (n = 5), B.1.1.28 (n = 8), B.1.1.33 (n = 1) and P.2 (n = 1); January 2021: B.1 (n = 1), B.1.1.28 (n = 4), P.2 (n = 9) and P.1 (n = 1); February 2021: B.1.1.143 (n = 1), B.1.1.28 (n = 2) and P.1 (n = 24).

## Specimens’ SARS-CoV-2 PCR cycle threshold values evolution over time

**Figure 3:**
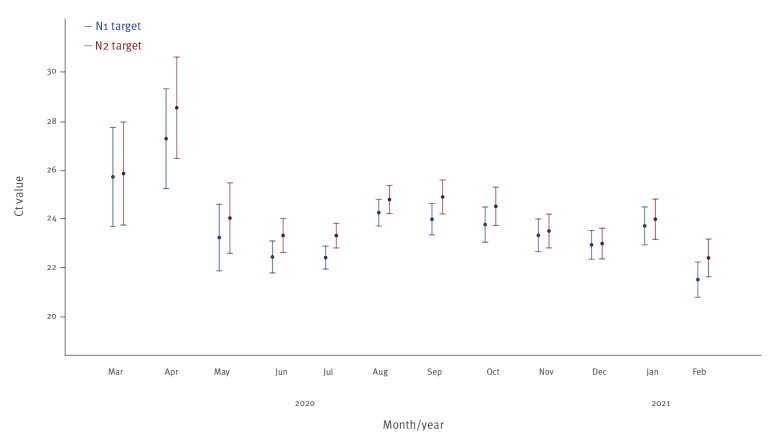
Cycle threshold (Ct) mean values and confidence interval of N1 and N2 targets in RT-qPCR performed at a tertiary hospital in Porto Alegre, Southern Brazil, March 2020–February 2021 (n = 3,868 specimens)

We also obtained the Ct values of the two genes of the nucleocapsid protein, N1 and N2 [3] from all cases in our hospital during the time of our investigation (n = 3,868 specimens). Month by month comparisons across the 12-month study period were conducted. Ct values in February 2021 were the lowest observed in the period (mean: 21.11; 95% confidence interval (CI): 20.39–21.84; p = 0.14; and mean: 22.01; 95%CI: 21.23–22.78; p < 0.001, for N1 and N2 targets, respectively; one-way analysis of variance (ANOVA); Figure 3) when P.1 lineage was detected in 24 of 27 specimens.

Ct: cycle threshold.

## Discussion

RS state has a population of 11,422,973 and land borders with Uruguay and Argentina. RS was one of the least affected Brazilian states during the first COVID-19 pandemic wave (early July–September 2020), although the number of cases increased markedly from early November to late December 2020 [1]. A previous study conducted in the municipality of Esteio, RS, from May to October 2020 revealed that the most common lineages were B.1.1.33 and P.2 (initially called B.1.1.248) [5]. Genomic surveillance by the central state laboratory confirmed the predominance of these lineages across RS, following a distribution similar to that observed in other Brazilian states, but revealed that lineage P.2, first detected in October 2020, had been gradually replacing these as the predominant variant as of 31 January 2021 [6]. Lineage P.1 was first detected in RS state in a single clinical specimen obtained on 01 February 2021 from a resident of Gramado, a popular tourist destination in RS, who however, had no history of travel or contact with returning travellers from northern Brazil [2]. Whole genome sequencing in the current study detected a specimen with SARS-CoV-2 P.1 lineage from January 2021. It is therefore possible that P.1 variant was circulating in RS before February 2021, though, to date, there are no other published studies to our knowledge confirming this.

Until January 2021, the distribution of SARS-CoV-2 lineages observed in cases treated at our COVID-19 referral centre was similar to that observed in regional reports, which tended to follow the distribution of most Brazilian regions, except for the North, where lineage P.1 had been predominant since December 2020 [6,7]. In February 2021, however, an overwhelming increase in hospitalisations was observed from epidemiological week 6, temporally coinciding with the finding that lineage P.1 became predominant, accounting for the vast majority of specimens selected for genotyping in this period, albeit the number of specimens taken was small.

P.1 and P.2 lineages are descendants from B.1.1.28 but phylogenetic analysis has revealed that both emerged from independent evolutionary events [8]. P.1 has been termed VOC due to important mutations in the spike protein (K417T, E484K, and N501Y) some of which are found in B.1.351 (South Africa) and B.1.1.7 (United Kingdom) lineages. These mutations have been associated with higher transmissibility as well as a potential to promote reinfection [9]. Lineages harbouring the E484K mutation, such as P.2, are considered variant of interest because of their potential ability to escape from the immune system and their spread should also be monitored [8-10].

Since the hospitalisation dynamics observed in RS state resemble those noted in December in the northern states of Brazil, where P.1 vastly predominated among tested specimens, we hypothesise that P.1 might be at least partially associated with the striking 3.8-times rise in hospitalisations in RS. Previous findings suggesting that P.1 may be more transmissible than other lineages corroborate our hypothesis [7,9,11].

Regarding measures to control SARS-CoV-2 spread, in May 2020, the RS government implemented a system that divided the state into healthcare regions and, on a weekly basis, provided one of four possible classifications for each of such regions, thereby symbolising the risk of transmission. Each classification implied a lower or greater degree of restrictions in social and commercial activities. These included for example incremental reduction of capacity (e.g. hotels) of businesses or their types of provided services (e.g. serving on premises vs take away/delivery for restaurants) or opening hours – with closure of non-essential activities for the highest-risk-related classification. However, none of these measures limited urban or inter-cities mobility [12,13]. Furthermore, the system relied heavily on hospital capacity (i.e. number of available beds mostly in intensive care units) rather than on community transmission data. In addition, the rules for the restrictions were loosened over time, ultimately resulting in an absence of strict control measures for COVID-19 community transmission during the whole pandemic period. From May 2020 until the present date there was no major change in levels of physical distancing [12]. All these elements might have contributed to the dissemination of a more transmissible SARS-CoV-2 VOC.

Our data have limitations and must be interpreted accordingly. First, our sample comprises cases from a single referral centre, and the specimens were chosen according to convenience; thus, the study should not be interpreted as a prevalence investigation for our region. Nonetheless, it should be noted that the criteria for specimen selection of isolates from January (one P.1) and February 2021 (24 P.1) were the same. Second, although consistent with previous findings [9,11], the lower Ct values observed in February must be interpreted with caution, because Ct values were not adjusted for time from onset of symptoms.

## Conclusions

In summary, our findings raise concerns regarding a possible association between lineage P.1 and rapid growth in cases and hospitalisations. Despite the relatively low number of specimens analysed, the finding that the previously undetected P.1 lineage now accounts for almost nine in 10 specimens from a COVID-19 referral hospital in a state where exponential growth in hospitalisations has been observed warrants close attention and highlights the need for VOC surveillance and measures to control the spread. Further studies are necessary to investigate the potential association of P.1 dissemination with rapid increase in hospitalisations.

## Supplementary Data

310000 Supplementary Material
