# Supplementary material for: Detection of SARS-CoV-2 lineage P.1 in patients from a region with exponentially increasing hospitalisation rate, February 2021, Rio Grande do Sul, Southern Brazil
Source: Euro Surveill. 2021 Mar 25;26(12):2100276. doi: 10.2807/1560-7917.ES.2021.26.12.2100276 (PMC7995561; doi:10.2807/1560-7917.ES.2021.26.12.2100276)
Supplement: Supplementary Material [file 2100276_Supplementary_Table.pdf]

"This supplementary material is hosted by Eurosurveillance as supporting information alongside the article [Detection of SARS-CoV-2 lineage P.1 in patients from a region with exponentially increasing hospitalisation rate, February 2021, Rio Grande do Sul, Southern Brazil], on behalf of the authors, who remain responsible for the accuracy and appropriateness of the content. The same standards for ethics, copyright, attributions and permissions as for the article apply. Supplements are not edited by Eurosurveillance and the journal is not responsible for the maintenance of any links or email addresses provided therein."

Supplementary Table 1. Characteristics of sequenced specimens, setting of specimen recovery, and epidemiological history of patients.

Specimen	RT-PCR Date	Ct*		Lineage	Age groups	Setting	Travel or contact to traveler from Northern Brazil	GISAID ID (EPI_ISL_)
		(N1)	(N2)					
1	21/03/2020	13.15	11.90	B.1	80-89	Inpatient	N/A**	1163693
2	28/03/2020	16.76	15.44	B.1.1.161	40-49	Outpatient	N/A	1163694
3	30/03/2020	16.95	17.42	B.1.1.161	50-59	Outpatient	N/A	1163695
4	14/04/2020	17.60	17.80	B.1.1.161	50-59	Outpatient	N/A	1163697
5	30/04/2020	16.66	16.54	B.1.1.161	70-79	Inpatient	N/A	1163696
6	25/05/2020	15.46	14.91	B.1.1.161	30-39	Outpatient	N/A	1163698
7	25/05/2020	14.77	14.36	B.1.1.161	50-59	Inpatient	N/A	1163699
8	28/05/2020	18.52	19.30	B.1.1.106	40-49	Outpatient	N/A	1163700
9	04/06/2020	13.54	13.22	B.1.1.161	50-59	Outpatient	N/A	1163701
10	10/06/2020	12.30	12.81	B.1.1.161	50-59	Inpatient	N/A	1163702
11	13/07/2020	11.17	11.01	B.1.1.161	50-59	Inpatient	N/A	1163703
12	21/07/2020	13.80	13.10	B.1.1.28	60-69	Inpatient	N/A	1163704
13	30/07/2020	11.88	12.34	B.1.1.33	30-39	Inpatient	N/A	1163705
14	21/08/2020	14.83	15.09	B.1.1.161	40-49	Outpatient	N/A	1163706
15	26/08/2020	13.00	12.09	B.1.1.161	60-69	Outpatient	N/A	1163707
16	28/08/2020	12.89	12.51	B.1	60-69	Inpatient	N/A	1163708
17	04/09/2020	13.04	12.49	B.1.1.28	40-49	Outpatient	N/A	1163709
18	17/09/2020	14.49	14.02	B.1.1.28	30-39	Outpatient	N/A	1163710
19	29/09/2020	15.48	15.66	B.1.1.28	40-49	Outpatient	N/A	1163711
20	05/10/2020	15.40	14.30	B.1.1.161	30-39	Outpatient	N/A	1163712
21	29/10/2020	14.20	13.96	B.1.1.28	40-49	Outpatient	N/A	1163736
22	18/11/2020	22.65	24.93	B.1.1.28	30-39	Inpatient	N/A	1163713
23	18/11/2020	11.38	11.32	B.1.1.161	50-59	Outpatient	N/A	1163737
24	08/12/2020	13.82	13.14	P.2	40-49	Outpatient	N/A	1163714
25	09/12/2020	11.50	9.39	B.1.1.28	40-49	Outpatient	N/A	1163715
26	25/12/2020	12.75	12.55	B.1.1.28	50-59	Outpatient	N/A	1163716
27	03/01/2021	17.30	16.80	P.2	<2	Inpatient	N/A	1163532
28	07/01/2021	19.15	19.32	B.1.1.28	<2	Inpatient	N/A	1163532
29	09/01/2021	14.89	15.28	P.2	50-59	Inpatient	N/A	1163532
30	14/01/2021	14.86	15.17	B.1.1.28	<2	Inpatient	N/A	1163532
31	15/01/2021	15.26	14.15	P.2	30-39	Outpatient	N/A	1163532
32	15/01/2021	15.94	15.10	P.2	30-39	Outpatient	N/A	1163532
33	18/01/2021	15.52	12.50	P.2	30-39	Outpatient	N/A	1163532
34	20/01/2021	12.11	12.03	P.2	30-39	Inpatient	N/A	1163532
35	21/01/2021	16.07	15.91	B.1	20-29	Inpatient	N/A	1163532
36	22/01/2021	13.65	13.41	B.1.1.28	30-39	Outpatient	N/A	1163530
37	22/01/2021	15.04	15.48	P.2	<2	Inpatient	N/A	1163532
38	27/01/2021	10.98	9.80	P.2	20-29	Outpatient	N/A	1161403
39	28/01/2021	17.01	17.31	P.2	30-39	Outpatient	N/A	1161402

40	30/01/2021	14.37	13.24	B.1.1.28	<2	Inpatient	N/A	1161401
41	30/01/2021	12.49	11.99	P.1	20-29	Outpatient	Unknown	1133133
42	06/02/2021	11.42	11.08	P.1	<2	Inpatient	No	1133132
43	08/02/2021	19.90	19.97	P.1	<2	Inpatient	No	1133131
44	08/02/2021	12.03	12.41	B.1.1.28	30-39	Outpatient	N/A	1161415
45	10/02/2021	17.99	19.61	P.1	40-49	Outpatient	Unknown	1133120
46	10/02/2021	16.26	16.88	P.1	20-29	Outpatient	No	1133128
47	10/02/2021	14.21	16.33	P.1	20-29	Outpatient	Yes	1133129
48	10/02/2021	11.79	11.35	P.1	60-69	Inpatient	No	1133130
49	11/02/2021	11.56	13.14	P.1	20-29	Outpatient	No	1133137
50	13/02/2021	17.91	17.48	P.1	40-49	Outpatient	Yes	1133127
51	13/02/2021	18.24	19.23	B.1.1.143	60-69	Outpatient	N/A	1161413
52	14/02/2021	16.52	17.35	P.1	20-29	Inpatient	No	1133125
53	14/02/2021	12.57	12.82	P.1	20-29	Inpatient	No	1133126
54	15/02/2021	17.43	19.11	P.1	20-29	Outpatient	No	1133121
55	15/02/2021	17.14	17.85	P.1	20-29	Outpatient	No	1133124
56	18/02/2021	11.29	12.62	P.1	20-29	Outpatient	No	1133134
57	18/02/2021	12.73	14.55	P.1	30-39	Outpatient	No	1133123
58	18/02/2021	13.44	11.31	P.1	20-29	Outpatient	No	1133134
59	18/02/2021	21.77	24.62	P.1	20-29	Outpatient	Yes	1133141
60	19/02/2021	17.90	18.20	P.1	50-59	Outpatient	Yes	1133138
61	20/02/2021	16.96	16.60	P.1	30-39	Outpatient	No	1133139
62	22/02/2021	21.67	22.29	B.1.1.28	50-59	Inpatient	N/A	1161414
63	22/02/2021	10.81	10.38	P.1	50-59	Inpatient	No	1133136
64	22/02/2021	16.10	17.50	P.1	30-39	Outpatient	No	1133140
65	22/02/2021	12.67	12.51	P.1	50-59	Outpatient	No	1133142
66	22/02/2021	10.13	11.40	P.1	20-29	Inpatient	No	1133143
67	22/02/2021	17.70	18.90	P.1	30-39	Outpatient	No	1133144
68	23/02/2021	16.17	15.67	P.1	20-29	Outpatient	Yes	1133135

*Cycle threshold (Ct) values obtained by RT-qPCR assay using the two genes of the nucleocapsid protein, N1 and N2, as targets according to CDC/USA protocol.

**N/A = not applicable. Information regarding travel or contact to traveler from Northern Brazil was collected only for patients with the P.1 variant.

The total number of males in 2020, Jan 2021 and Feb 2021 were 10, 4 and 11, respectively. The number of males among the P.1 variant specimens were 11.
